# Social Work Practice in United Arab Emirates (UAE) Schools during and after the COVID-19 Pandemic: A Qualitative Study

**DOI:** 10.3390/ijerph21101323

**Published:** 2024-10-06

**Authors:** Abdulaziz Albrithen, Shamma Alfalasi

**Affiliations:** Department of Social Well-being, College of Humanities and Social Sciences, United Arab Emirates University, Al Ain 15551, United Arab Emirates; sh.alfalasy@uaeu.ac.ae

**Keywords:** social work, COVID-19 pandemic, school social worker, qualitative method, UAE

## Abstract

Besides their educational mission, public schools ground and support the health and psychological, emotional, and social development of students. The educational system is special because it combines multiple integrated responsibilities and duties that transcend the scope of any other social welfare program. Social workers are crucial constituents of educational systems and are expected to attend to the well-being of students while ensuring the effective performance of the educational system. Given such rigorous demands, this study assesses and compares the actual functions undertaken by social workers in schools in the United Arab Emirates during and after the COVID-19 pandemic. In so doing, it aims to help improve educational systems worldwide. The nuanced results of this study will better elucidate the crucial capabilities of social workers in the dynamic educational system and illuminate the challenges they confront during emergencies.

## 1. Introduction

Educational systems worldwide are responsible for imparting knowledge to younger generations as well as facilitating the psychological, emotional, and social development of the ever-changing collective global student body. As a key social welfare benefit, public schools also aim to guide the population’s development to enable youngsters to become productive members of society [1]. Social workers are routinely assigned to public schools to help ensure that such abstract goals are achieved. Scholars have largely underreported the responsibilities and expected contributions of social workers, particularly in the United Arab Emirates (UAE); therefore, this study investigates the actual circumstances pertaining to the roles enacted by social workers in public schools during and after the COVID-19 pandemic. In so doing, this study aims to deliver a nuanced and operational understanding of the challenges and strengths of social workers in schools so that policymaking organizations can become cognizant of the relevant key indicators and empirical expectations.

Education was one of the global social systems most affected by the COVID-19 pandemic. School staff, families, suppliers, publishers, and numerous other ancillary participants felt the massive impact of lockdowns and quarantine measures. These negative effects are still being assessed, and the comprehensive apprehension of the limitations that were overcome could take decades of study [2,3,4,5,6]. Nevertheless, a large gap persisted for decades before the pandemic in the understanding of the roles enacted by social workers in schools and their responsibilities toward students, staff, and parents. This lacuna became even more apparent during and after the COVID-19 pandemic, which could be considered a driving force for stakeholders to finally resolve this issue and establish an empirical and functional framework to help improve physical, emotional, and mental health policies in public education. Considering that the challenges that occurred represent lessons that should be learned from the crisis, an attempt should now be made to avoid any similar future circumstances. The heavy and incessant reliance of families and governments on limited educational social worker staff to provide real-time feedback on the organic human well-being fostered by the existing system during and after the pandemic is a significant issue for investigation [7,8]. Notably and unsurprisingly, the poorest of society’s members were also the most affected [7,8]. and clearly most needed the predominant amount of the resources and services of social workers.

COVID-19 expedited the development and implementation of new capabilities, methods, and technologies that have, perhaps, permanently changed staff training and classroom education environments. Mental health crises and individual accommodations have become more focal for policymakers than ever before. These priority changes and the general realization of social vulnerabilities have increased expectations from school social workers to deliver welfare services and simultaneously ensure dynamic policy enforcement [7,8]. The full scope of the impact of such functions on public schools remains unknown. Therefore, this study investigates the responsibilities discharged by social workers in UAE schools during and after the COVID-19 pandemic. In so doing, it seeks to contribute to the improvement of global public education systems in an empathetic and forward-thinking manner.

The effects of the COVID-19 pandemic count among the daily effects confronting social workers and include the school environment. Individuals, families, and governments are concerned about the negative impact of the pandemic on school functions. The most disadvantaged, vulnerable, and poorly served individuals, families, and communities are also the worst affected sections of populations across the world.

Like wars, financial crises, and other global diseases, the recent pandemic was a global phenomenon; nevertheless, local contexts, cultures, and knowledge are important. This study adopts the lens of social work practitioners across school settings as a significant and common area of social work practice to focus on how the UAE responded to the COVID-19 pandemic.

## 2. Emergent Literature

School social workers within the purview of the education system have always been concerned about addressing the well-being needs of children and families [9,10]. However, the profession has become dominated by individual and small-group special education services because of the remarkable development of social work practice in schools. Mental health services are also delivered to students within the confines of the school building and the traditional school day [11,12].

The COVID-19 pandemic caused unprecedented closures of education agencies in many countries worldwide. The shutdowns varied across countries but generally spanned from early 2020 to the end of 2022. As education systems closed, education professionals rushed to develop and implement online curricula and distance learning to meet the academic needs of societies. Diverse attempts were made to fulfill academic needs. Nevertheless, children and families confronted significant psychological, social, and economic challenges during the period of social distancing and societal closure [13].

Social work studies have suggested that the COVID-19 pandemic has become an academic endeavor since its outbreak in 2020. The literature has grown in the years following the outbreak of COVID-19 as researchers have contemplated the pandemic’s impact on daily life as well as the learning that could be imbibed to facilitate societies to better address future pandemics and disasters. The COVID-19 pandemic also triggered the discovery of alternative ways of working during the crisis, which could prove advantageous to the profession and its beneficiaries [14]. Such attempts have contributed to supporting the tributaries of the theoretical frameworks of the profession. This facet is a characteristic of social work; the applied aspects of this domain feed its theoretical scaffoldings. This characteristic contributes effectively to the development of the profession and reduces the gaps between theories and practices, which is a theme that preoccupies many individuals interested in the frameworks of social work.

In recent years, several global webinars on social work have highlighted the practical learning acquired during the two years of the pandemic (2020–2022). Social workers experimented with teleworking during such sessions, which delivered specific benefits such as the potential of facilitating greater contact with people who need certain types of services. However, webinar organizers also recognized several country-specific contexts of persisting exclusion because of the lack of access to technologies such as the Internet. Nevertheless, constructive insights have been obtained about the reduction in bureaucratic and rule-bound requirements encompassing usual practices [15]. Noteworthily, such solutions must be evaluated from the purview of aspects emphasized by the social work code of ethics.

The literature examining changes in school-based social work practices during the COVID-19 pandemic is growing. For example, much of the research conducted in the United States has focused on the characteristics of practices that transformed because of social distancing. Communication and interactions between school social workers and parents of students increased steadily and were centered around two main activities: food assistance and referrals to families and parent attendance and training [16,17]. One study delineated three principal themes: (a) the need for the professional development of teachers and support for student health, (b) concerns about the quality of available services, and (c) insufficient access to mental health professionals and programs in schools. Studies have also discussed inadequate access to mental health professionals and programs. A study based on the proportion of immigrant students in the school [18]. mooted concerns about the issue of inequalities imposed by the pandemic on the nature of social work practices in schools. The American ground realities indicated that school social workers had achieved appropriate levels of adaptation to their professional functions in the new practice contexts inflicted on them by the pandemic, for example, social distancing [19,20]. However, the factual circumstances also revealed significant professional pressures faced by social workers in American schools apart from personal challenges that hindered the effective fulfillment of their functions as professional social workers. American school social workers reported significant concerns about their ability to successfully deliver professional services because of low student motivation and lack of engagement in online learning. Besides their significant concerns about student underperformance in the early months of the pandemic [20], U.S. social workers also noted the significant unmet basic needs of children and families, including food, health care, and housing. Poor mental health and poverty increased pandemic-related difficulties, which are linked to the sociodemographic compositions of schools. Student engagement with social services was also significantly lower during lockdowns than prepandemic levels [21], probably because of unsatisfied material needs and reduced face-to-face contact between social workers and their clients.

The relevant literature on the U.K. has highlighted concerns about the nature of contact between social workers and school children during the COVID-19 pandemic [22]. It has also illuminated the characteristics of school social work practices, emphasizing improvisation and creativity in the praxes adopted by social workers in the face of massive disruption and emergency conditions [23]. Scholars in Romania have attempted to explore the pros and cons of online school social work processes. They have identified the most significant challenges of remote social work methods, such as building trust and safety, confronting ethical challenges, and ensuring quality and efficiency [24]. Such research endeavors could envisage future patterns of professional practices, namely, hybrid social work methodologies (traditional and digital). A study conducted in Portugal evaluated responses extended during the pandemic and noted how emergency plans and challenges were actively integrated through information and communication technologies [25]. Researchers in Finland probed the experiences of social workers in expanding the use of digitally mediated social work while working with clients during the first wave of the pandemic. The qualitative analyses offered by social workers revealed that their practices were purely digitally mediated; however, they relied substantively on the judgment and expertise of social workers [26]. In the same context, the Norwegian experience revealed that the digital competence required of social workers included technical skills and content creation as well as abilities to promote dialogue and encourage problem-solving [27]. As we surpass the pandemic, it will become more appropriate to shift away from discussing the digital competence of individual social workers or their resistance to digital learning and move toward strengthening the enabling conditions of digital social work. The education and advanced training of social workers represent fundamental requirements for social work practices in schools to become more effective. The Greek experience revealed a significant increase in behavioral, mental health, and financial difficulties for students and their families and evidenced insufficient means and sources of assistance. Scholars have also discussed the limited practical preparedness of school social workers. In addition, Greece’s experiences indicate that the pandemic period could offer school social workers the opportunity to reconsider their professional intervention models. More effective models could be devised to tackle the complexities and durations of problems created by the pandemic for both social work professionals and their clients. Such new models could result in the progression of school social work in Greece [28].

Much of the literature on school social work accomplished during the pandemic has focused on the roles of social workers, their perspectives on family engagement, their behavioral difficulties, their patterns of family engagement, and the challenges associated with the nature of their engagement. Addressing basic needs, providing resource referrals, and assisting with the emotional and psychological needs of children and families have been reported as some predominant tasks of professional work conducted during that period. The literature reveals how social workers were able to implement and attempt to engage families, a professional orientation described in the standards for school social work practice [29] while maintaining the ethical boundaries posited in the social work code of ethics [30].

The present study is the first to discuss the professional practices of social workers in UAE schools. It also compares the roles they enacted during and after the COVID-19 pandemic and reveals the positive outcomes and difficulties that essentially affected professional social work in schools directly as a result of the global health crisis.

## 3. School Social Work in the UAE

The school social work program denotes the UAE’s largest employer of social workers. It was established in 1972 when the Ministry of Education appointed Egyptian social workers to serve in Emirati schools [31]. This section discusses the history, models, responsibilities, benefits, and problems encountered in the school environment in the UAE.

### 3.1. Historical Overview

Social work originated in the United States and was thereafter quickly adopted by Egypt [32]. The U.S. model focused on family welfare but the Egyptian model concentrated on schooling. Sowa and Vega [33] indicated that improvements in the educational system in the UAE were driven by the discovery of oil, which enabled programs to be rebuilt from the ground up. Initial efforts to this end were made within the framework of UAE University’s Sociology Department, which became an independent section in 1995 and was accorded an expanded scope that included Emirati society and its value system. Hence, the UAE model introduced changes that distinguished it from the guidelines established in the United States and Egypt.

### 3.2. Social Work Practice Models

The UAE has two basic social work models: family–school cooperation and strengths-based practice.

#### 3.2.1. Family–School Cooperation Model

Social workers promote the active engagement of schools as well as families in the educational welfare of children. To this end, they organize meetings, workshops, and related activities to foster collaborations and goal-setting. Families directly receive additional services to facilitate their cooperation and help them address challenges. Such enabling actions involve intervention and advocacy within the school system. Empowerment is pivotal to the development of robust partnerships between families and schools in the UAE [34].

#### 3.2.2. Strengths-Based Practice Model

This model is facilitated by social workers and identifies the inherent strengths and assets of students, families, and communities. The processes posited in this model allow all stakeholders to amplify their strengths. Thus, all parties can achieve positive outcomes, minimize weaknesses and deficits, and foster the necessary resilience to allot time and energy to skills development and personal growth. Participants work collaboratively on these aspects and learn naturally how to help each other, a key feature of maintaining self-efficacy and confidence [35].

### 3.3. School Social Worker Responsibilities

Most school social worker roles in the UAE have been previously described in this paper. Some related resources [33,36] have overviewed these responsibilities as

- Facilitating effective communication (collaborations) between schools, families, and communities
- Providing family services and allied assistance
- Intervening in situations involving a child’s social, emotional, and/or behavioral development
- Counseling students, families, and staff members
- Connecting participants to external community resources, agency programs, and academic principles

### 3.4. School Social Worker Recruitment

School administrators and relevant educational authorities supervise the recruitment of social workers in the UAE, using a fair and amenable hiring system that aims to ensure the hiring of qualified, licensed, and available candidates [37].

### 3.5. Social Worker Benefits

School social workers can participate in several professional development opportunities including attending workshops, training programs, and conferences that aim to enhance their skills and knowledge. The salaries of school social workers are generally based on their experience levels and qualifications as well as the type of employing educational institution. Overarching hiring and payment policies are more directly influenced by government regulations and market demand.

Many UAE educational institutions offer their social workers healthcare benefits and hiring institutions are expected to promote the work–life balance of social workers by offering them flexible working hours, vacations, and other benefits that support their overall well-being.

### 3.6. UAE Laws and School Social Worker Benefits

Federal Law No. 3 of 2016 (Wadeema’s Law) requires schools to report cases of suspected child sexual abuse to law enforcement. School social workers form the cornerstone of this mandate; they are expected to increase overall awareness, ensure due diligence, and attain specialized training on recognizing, reporting, and supporting victims. Educational institutions should empower their social workers and other staff members by implementing tailored child protection policies to facilitate compliance with national statutes [38].

### 3.7. Challenges and Dilemmas

Social workers in UAE schools often encounter difficulties because of limited resources and heavy caseloads that can hamper the quality and effectiveness of their services. The lack of training of school social workers in specific areas such as career counseling or mental health can result in dilemmas in addressing the diverse needs of students. In addition, school social workers often experience difficulties associated with cultural sensitivities and norms, which require them to adapt their approaches to individuals. All school employees, particularly staff members designated as social workers, experience the challenge of effectively coordinating and collaborating with multiple stakeholders and simultaneously focusing on their personal well-being [39].

School social workers also occasionally find it difficult to communicate effectively with stakeholders because of language barriers, cultural differences, or trust-related deficiencies. Indeed, discrete factors could explain the seeming unwillingness of many students and parents to engage with social workers. This resistance further complicates the professional expectations of school social workers, who must establish and maintain delicate boundaries and institute careful and deliberate protocols in individually building rapport with all the concerned parties.

## 4. Methods

Qualitative studies tend to be interpretive and aim to intensively understand phenomena within their contexts. Generally, qualitative data are collected from the point of view of participants to elucidate situations, relationships, and characteristics of social practices [40,41]. Therefore, the qualitative methodology is best suited for the present study given its aim to elucidate the state of social work practice during and after the COVID-19 pandemic.

The interview questions followed a general plan; therefore, the researchers decided to use semistructured interviews, a form of querying that blends unstructured and structured discourses. The interview questions were developed, structured, validated, and piloted before the interviews were held but the interviewers did not need to follow any particular phrasing or order. Field testing has revealed that this type of interview is open-ended, allows flexibility, but simultaneously adheres to an orderly and predetermined thematic framework.

We conducted direct in-depth interviews with UAE school social workers (n = 22) to achieve a comprehensive understanding of their changing responsibilities during and after the COVID-19 pandemic. We adopted a field-of-practice approach and asked them nine questions on the identification and synthesis of their roles and functions. The questions were designed based on the study objectives. Pilot interviews were conducted and their outcomes revealed that the following queries were the most apt for the study:

1. What is the general nature of the basic responsibilities you performed in the school during the COVID-19 pandemic? Please provide details in terms of your (a) administrative responsibilities, (b) professional responsibilities, and (c) recreational activities you organized.
2. How did you implement your roles as a school social worker during the COVID-19 pandemic? Please provide details in terms of (a) administrative responsibilities, (b) professional responsibilities, and (c) recreational activities you organized.
3. Have any changes occurred after the COVID-19 pandemic in your functions as a school social worker? How did your roles change or remain unaltered? Please discuss this aspect in detail in terms of your (a) administrative responsibilities, (b) professional responsibilities, and (c) recreational activities you organized.
4. Do you believe that your service delivery with respect to the roles you played as a school social worker was more effective before or during the COVID-19 pandemic? Why?
5. Do you think the COVID-19 pandemic affected social work practices in schools?
6. What difficulties did you encounter in delivering varied services to students and parents during the COVID-19 pandemic?
7. Do you think school social workers were sufficiently competent to provide students with remote services during the COVID-19 pandemic?
8. Could you suggest how the overall effectiveness of school social workers can be enhanced?
9. Could you suggest how the effectiveness of school social workers can be increased in future crises such as the COVID-19 pandemic?

The interview data were manually processed and analyzed with support from NVivo 10 for Windows SP6 Readme program, which allows the detailed coding of interview transcripts and document texts to identify emergent themes and subthemes related to descriptive and procedural concepts. It then enables “coding on” to identify the characteristics and dimensions of obtained concepts.

Immediately after each interview was completed, the researchers wrote a one-page interview memo encompassing three key sections. The first section listed moments of the interview that the researchers deemed significant and included notes on the facial expressions, gestures, and tone of voice of the respondent. The second section contained analytical notes on how the interview fulfilled the study objectives and ensured complete answers for each question. The third section considered the methodological and ethical issues evoked by the interview session and the manner in which it was conducted. This interview memo facilitated the critical thinking of the researchers vis-à-vis their positionality and reflexivity [42,43].

The interviews occurred at private and government schools in Al Ain City. All of the interviewed school social workers were UAE citizens who were recruited via a formal letter addressed to the schools. The participation of all respondents was voluntary. Every interview lasted between 60 and 80 min and was conducted face-to-face in a quiet and secluded space on a specific date and time. Four of the 22 participants were male and 18 were female. The participants were aged between 25 and 42 years and had accrued professional experience ranging from 5 to 19 years.

## 5. Results

The study results are discussed according to the three types of interview questions: practices followed during the pandemic, routines after the pandemic, and general challenges resulting from the pandemic.

### 5.1. Social Work Practices Adopted during the Pandemic

#### 5.1.1. Administrative Responsibilities

The COVID-19 pandemic diminished the positive energy of social workers because their interactions with students, teachers, administrators, and parents became restricted. Most of their activities were conducted on remote platforms such as WhatsApp (version 2.23.12.75, Android OS 5.0), Zoom (version 5.17.11), and Teams (version 23335.208.2601.834), which yielded scant benefits in terms of their human-factor expectations. Notably, students were far less influenced by remote PowerPoint presentations than by interactive in-person sessions.

This finding reveals that the administrative responsibilities performed by social workers during the pandemic were limited by technology tools.

Nonverbal communication skills based on aspects such as eye contact and body language are critical in social work, especially during interchanges conducted for guidance related to well-being and mental health. However, the remote tools adopted during the COVID-19 pandemic were extremely raw and offered restricted capabilities because of insufficiencies in national and worldwide infrastructure. For example, social workers found it extremely frustrating that they were forced to depend on even more physically distanced modes of intervention in attempting to address frequent absences by students. Before the pandemic, school social workers would engage more directly with students and their family members at conferences or in their homes, among other means. Similar scenarios affected the ability of social workers to interact with teachers and other staff members. The interviews conducted with the participating school social workers revealed that their administrative practices during the pandemic crisis were inefficient.

In general, the administrative responsibilities and roles of school social workers changed during and after the pandemic, which became focal to their functioning because of the challenges it posed and the outcomes it generated.

#### 5.1.2. Professional Responsibilities

The access of school social workers to personal and private information was affected during the pandemic when they were compelled to work remotely or from home. In addition, their intervention-based duties were substantially restricted because they had to discard their key interactive toolbox; they could not achieve eye contact and were significantly restricted in tendering empathetic bodily and voice expressions. Overall, their abilities to see, interpret, and diagnose hidden signals became unavailable. The interviewed social workers indicated their inability to deploy the skills they had studied and practiced during the prepandemic years. The most prominent of these skills entailed effective communication with clients along with other abilities often associated with the strength of direct communication with clients (e.g., persuasion, negotiation, and influence).

Reflecting on this experience, an interview participant [SW11] stated, “Unfortunately, during my case study sessions with students and holding the first session to introduce them to the role of the social worker, it was done behind a phone, which, of course, reflected negatively because I needed at least three sessions with the student and their parent through social media platforms to build a professional relationship with them, instill confidence, and assure them that the information is within the framework of confidentiality as long as it does not pose a danger or harm to any members of the community in the surrounding environment”.

Another respondent [SW17] asserted, “I couldn’t apply any of the diagnostic tools I had previously used, such as standardized scales or even simple scales”.

Social workers are also expected to engage directly with families by implementing treatment plans and assigning tasks to accomplish between sessions. A typical behavioral intervention process for students would adhere to the following guidelines:

- Gathering the necessary information
- Analyzing the behavioral and psychological issues of students based on the gathered observations by teachers and school reports
- Conducting initial assessments, identifying possible causes, and honing the list of potential tools
- Developing individualized treatment plans including specific strategies and activities to address behavioral difficulties and inculcate constructive changes
- Implementing treatment plans through counseling sessions and therapy, including stipulating family activities and tasks
- Monitoring the progress of students and family members during the implementation of treatment plans
- Conducting periodic follow-up sessions

Evidently, school social workers found it extremely difficult to achieve satisfactory results during the pandemic, especially when families also experienced varied forms of trauma including the curtailing of their ability to commiserate in person.

In general, almost all of the interviewed social workers agreed that they confronted great difficulties in adapting their professional roles and functions during the pandemic, especially during the first months of the novel coronavirus outbreak. However, they began to adapt better after six months. Their professional roles were also focused on creating adaptations for students and families. The interviewed social workers confirmed that children and their family members predominantly displayed stress about contracting the virus and experienced anxieties caused by the possibility of obtaining vaccines.

The interviews generally indicated that the effectiveness of social work practice declined significantly during the recent pandemic. Specialists were preoccupied with problems related to the weak professional application of social work during the 1970s. Such concerns were recently resurrected because of the circumstances imposed on social workers by the global health crisis.

#### 5.1.3. Recreational Activities

According to an interview participant [SW2], “As school social workers, we had to adjust the recreational activities that we wanted to introduce to students and involve them in by organizing and selecting suitable parties based on the health and social conditions in the virtual school environment”.

To remotely provide and facilitate such activities during the pandemic, school social workers reported engaging in the following activities:

- Conducting online workshops using live video platforms to enhance social and emotional skills
- Implementing online competitions, games, and educational challenges
- Conducting counseling sessions via phone and/or video
- Organizing developmental events such as online seminars and skills training
- Disseminating awareness campaigns via social media
- Taking virtual field trips (e.g., through museums or recorded videos)

The interviewed social workers almost unanimously articulated the opinion that recreational activities did not achieve the desired interaction levels. However, such recreational or professional activities are considered aspects of social work.

### 5.2. Social Work Practices Adopted after the Pandemic

#### 5.2.1. Administrative Responsibilities

The interviewees identified the following changes occurring after the pandemic as the most significant.

First, social distancing requirements persisted and remained severely restrictive despite the loosening of restrictions and resumption of personal interactions. People continued to rely on phone calls and online sessions.

Second, students and family members experienced an increased need for mental health support because of the impact of the pandemic on the psychological well-being of most students. The long-term isolation due to the lockdown demanded the creation of new individual consultation protocols, which mandated the institution of online policies and procedures.

Third, the social and psychological needs of students became more pronounced as many schools adopted remote learning and other advanced technologies at the end of the pandemic. However, most psychosocial support was delivered online as all client parties were forced to improve their ability to acquire and operate key social media and conference platforms such as Zoom and Teams.

Fourth, by the end of the pandemic, school social workers had constructed extremely effective infrastructure and personal networks connecting families and schools. Therefore, social workers were made responsible for COVID-19 postpandemic awareness drives and were tasked with organizing campaigns on maintaining mental health and community safety.

Fifth, collaborations forged by school social workers with external entities such as hospitals and health centers increased because the school social workers were increasingly tasked with supporting students and family members who needed additional services.

Overall, it could be stated that the administrative responsibilities of social workers have gradually normalized to approximately the same duties as before the pandemic.

#### 5.2.2. Professional Responsibilities

The pandemic exerted a significant impact on the professional responsibilities of school social workers. An interviewee stated [SW6], “I faced challenges and difficulties when opening student files for case studies, diagnosis, and providing appropriate treatment”. Social workers in UAE schools recounted experiencing the following changes:

- Existing tools required to be adapted to encompass more wide-ranging services and consultations including the initial data compilation because many educational activities were offered remotely
- It became more challenging to extend social and psychological support online
- Mental health difficulties increased in students and families, and the duties of social workers were accordingly amplified
- Case follow-up tasks could be expedited using social media but the interactive complexities remained challenging because social workers have long depended on nonverbal elements of human expressive elements, which were curtailed by remote interactions
- Increasing community awareness of postpandemic mental health issues became paramount for school social workers, who were tasked to further guide students and families on how they could cope with the stress and anxieties caused by continued social distancing and the persistent emphasis on remote learning

One interviewed social worker [SW8] opined: “The postpandemic period is like the waves left behind by a large ship after crossing the sea. Although the ship has passed, the ripples of the water remain for a while, and this is what happened with our professional practice during the postpandemic period”.

#### 5.2.3. Recreational Activities

The participants reported that they took some time to become adept at helping their clients regain their cognitive abilities and readjust to social life in the postpandemic environment. Most recreational interactions undertaken during the initial postpandemic period were focused on social communication, disease prevention, and adaptation to new physical contact policies. One participant stated [SW14]: “I faced significant challenges in organizing school trips, with very few students registering due to their ongoing fear of infection and their desire to continue isolation from the external community to avoid any interaction. Here, the changes in the nature of my work became apparent”.

In general, the majority of social workers disclosed attempts they made to restore the activities they used to perform before the pandemic, which helped them enjoy the educational environment of the school. The pandemic period has been surpassed; therefore, any persisting effects of the pandemic will hopefully dissipate completely in due course.

### 5.3. General Challenges Resulting from the COVID-19 Pandemic

Overall, the pandemic amplified the need for psychological and social support and thus added to the burdens shouldered by school social workers. Indirect interactions increased immediately after the pandemic, but direct communication also became more commonplace even though it remained restrictive. Rare opportunities to organize mass recreational and social activities in schools were deemed more effective in the prepandemic and postpandemic periods. The psychological and social challenges produced by the effects of the pandemic gradually became logistically and technologically less difficult to maneuver. An interviewee [SW19] reported: “During the COVID-19 pandemic, we faced many challenges. The distance of students from teachers and social workers reduced student commitment and revealed neglect in some families. Some students were absent or sleeping during lessons. When communicating with parents, we encountered unexpected excuses and justifications. Additionally, new and undesirable behaviors emerged, such as disruptions during virtual classes, students expelling each other from online classrooms, transferring assignments, and seeking external assistance in exams. Some problems were overcome through collaboration with parents although achieving desired solutions was sometimes hindered by obstacles like student or family COVID-19 infections. The flexibility of pandemic-specific behavioral regulations prevented the achievement of required solutions, which needed to be more stringent”.

Almost all of the interviewed social workers agreed that it was difficult at the outset to adapt to the pandemic-induced changes. This initial problematic period was followed by the difficulty of helping students and families adapt to the initiated changes. Most of the respondent social workers also indicated competence-related challenges of professional social work applications because of the circumstances and barriers resulting from the novel coronavirus crisis. Most social workers could not use their acquired skills and tools and could not apply their learned methods and tactics that would help them in the performance of their professional roles and functions. Moreover, some diseases unknown to the school social workers became observed in their domains. These diseases generally represented psychological disorders and posed difficulties for school social workers who were not sufficiently experienced to deal with such problems. Further, no evidence-based, scientifically tested, and practical guidelines existed to help school social workers devise effective professional applications.

In broad terms, the respondents identified the following two difficulties in the discharge of their tasks during and after the pandemic.

First, challenges were confronted in the delivery of assistance. Social and psychological interventions required personal and physical interactions, which were difficult when such services were remotely delivered

Social workers had to adapt to new technology. The school social workers were unaccustomed to the extensive use of technologies for interactive communications and found that the characteristics and membership of their field changed dramatically because of COVID-19.

## 6. Conclusions

The 22 interviews conducted for this study revealed that school social workers in the UAE confronted extremely challenging tasks and shouldered unexpected responsibilities during the recent pandemic. The COVID-19 crisis clearly highlighted the crucial roles enacted by social workers in microsocieties. The data synthesis performed for this study disclosed that the tasks performed by school social workers during the pandemic were focused largely on educating and encouraging students and reassuring them. This finding aligns with the results reported by another study [18]. Also, the present study highlights the performance of additional social work tasks such as following up with families to achieve the desired outcomes using only remote means. This result is consistent with the outcomes of other related investigations conducted by Karagkounis et al. [28]; Sugrue et al. [16], and Daftary et al. [17]. Interestingly, physical distancing protocols caused several problems that social workers usually faced to decline, for instance, fights and antisocial behaviors among students. The following points summarize the overall challenges confronting school social workers during and after the pandemic:

- Technical limitations and weak Internet connectivity caused difficulties in delivering online services and adversely affected the quality of social worker interactions and support
- The absence of direct communication with students and families reduced the ability of social workers to provide personal support and apprehend the nuances of cases
- The need to extend psychological and social support for students was amplified because of the effects exerted on their mental health by the pandemic, which increased the professional burden on school social workers
- Social workers were required to comply with health and safety measures in delivering personal services, which often caused increased stress to all concerned parties
- The negative economic impact of the pandemic further increased the social support needs of families

Other international studies have also discussed these challenges [20,21,22,24,26,28]. The pandemic placed extreme demands on the profession of social work, and the interviews conducted for this study with UAE school social workers highlight the importance of the vibrant and resilient personality this profession requires. Studies conducted in other countries have also illustrated the need for resilience [23,25]. Technical competencies in the use of electronic tools are essential but such abilities cannot sufficiently ensure the delivery of quality social services. The capabilities of social workers to handle pressure from multiple sources, tackle often conflicting social cultures, and willingly develop skills and competencies to overcome technical and logistical difficulties testify to their personalities. Many social workers exhibit the untiring inclination to overcome challenges, no matter how severe, and display larger-than-life efforts to support the rest of humanity. Other studies have also described the difficulties encountered by social workers in discharging their professional roles and functions during the COVID-19 crisis [19,20,28]. In sum, school social work denotes a profession that was significantly adversely affected by the COVID-19 pandemic in the UAE as well as globally.

This paper documents the challenges and tensions confronting social workers in UAE schools during and after the COVID-19 pandemic. The most prominent of these difficulties concerned the remote delivery of professional and administrative services. Other social work-related issues that need further research and exploration are evident. First, more research initiatives must probe the relevant challenges and responses in discrete areas of social work applications in the context of the pandemic. Second, the social work and academic communities must construct more wide-ranging discourses on the development and use of communication technology tools for social work applications. Third, as many of the studies examined have indicated, the pandemic represents an opportunity to enable school social workers in the UAE to recognize and tackle similar challenges in the future. Fourth, models of professional intervention can be reenvisaged and scholarly contemplation can be extended toward the construction of more effective approaches based on scientific evidence. Finally, scholars should also attend to how equality can be ensured and ethical professional praxes can be sustained.

## Data Availability

Data supporting this study are included within the article.
